# The Use of Berlin Heart EXCOR VAD in Children Less than 10 kg: A Single Center Experience

**DOI:** 10.3389/fphys.2016.00614

**Published:** 2016-12-06

**Authors:** Arianna Di Molfetta, Fabrizio Gandolfo, Sergio Filippelli, Gianluigi Perri, Luca Di Chiara, Roberta Iacobelli, Rachele Adorisio, Isabella Favia, Alessandra Rizza, Giuseppina Testa, Matteo Di Nardo, Antonio Amodeo

**Affiliations:** Department of Pediatric Cardiology and Cardiac Surgery, Pediatric Hospital Bambino GesùRome, Italy

**Keywords:** pediatric VAD, low weight LVAD, Berlin Heart

## Abstract

**Objective:** Despite the improvement in ventricular assist device (VAD) therapy in adults and in adolescents, in infant population only Berlin Heart EXCOR (BHE) is licensed as long term VAD to bridge children to Heart Transplantation (HTx). Particularly demanding in terms of morbidity and mortality are smallest patients namely the ones implanted in the first year of life or with a lower body surface area. This work aims at retrospective reviewing a single center experience in using BHE in children with a body weight under 10 kg.

**Methods:** Data of all pediatric patients under 10 kg undergoing BHE implantation in our institution from March 2002 to March 2016 were retrospectively reviewed.

**Results:** Of the 30 patients enrolled in the study, 53% were male, 87% were affected by a dilated cardiomyopathy with an average weight and age at the implantation of 6.75 ± 2.16 Kg and 11.57 ± 10.12 months, respectively. Three patients (10%) required a BIVAD implantation. After the implantation, 7 patients (23%) required re-intervention for bleeding and 9 patients (30%) experienced BHE cannulas infection. A total of 56 BHE pump were changed for thrombus formation (1.86 BHE pump for patient). The average duration of VAD support was 132.8 ± 94.4 days. Twenty patients (67%) were successfully transplanted and 10 patients (33%) died: 7 for major neurological complication and 3 for sepsis.

**Conclusion:** Mechanical support in smaller children with end stage heart failure is an effective strategy for bridging patients to HTx. The need for BIVAD was relegated, in the last years, only to restrictive cardiomiopathy. Further efforts are required in small infants to improve anticoagulation strategy to reduce neurological events and BHE pump changes.

## Introduction

The definitive treatment for the end stage pediatric heart failure is the orthotopic heart transplantation (OHTx). However, because of the lack of organ donors, especially in very small children, the use of ventricular assist device (VAD) is a validated therapy to bridge patients to the OHTx with a success rate up to 84% (Ibrahim et al., 2000; Reinhartz et al., 2003; Chang and McKenzie, 2005; Stiller et al., 2005; Hetzer et al., 2006; Gandhi et al., 2008; Brancaccio et al., 2010, 2013; Potapov et al., 2011; Almond et al., 2013; Miera et al., 2014; Kirklin, 2015; Mascio, 2015; Sandica et al., 2016).

The use of left VADs (LVAD) could successfully support patients till the OHTx, increasing the cardiac output (CO), unloading the left ventricle (LV) and decreasing the left atrial pressure and the pulmonary arterial pressure. The LVAD decrease the LV volumes and the LV work because of the flow distribution between the native LV and the LVAD (Barbone et al., 2001). In fact, in the presence of the LVAD, the total cardiac output is the sum of the LV output and the LVAD output. Increasing the LVAD cardiac output (increasing the LVAD speed in the continuous flow LVAD or increasing the pump rate in pulsatility flow LVAD) it is possible to obtain an increment of the total cardiac output (untill a determined plateau) and a higher unloading of the LV with lower LV volumes, lower left atrial pressure, lower pulmonary pressure and lower LV output. In the case of total support, all the cardiac output is totally provided by the LVAD and the aortic valve remains closed. This condition is usually avoided to permit to the LV at least a slight ejection and to the aortic valve to open at least every three cardiac cycle.

The effects of the LVAD on the right ventricular function is controversial. In fact, one of the major complications in LVAD patients is the right ventricular (RV) failure with an incidence among 13–44% in adults and an incidence of 42% in children (Chen et al., 1996; Kukucka et al., 2011; Saraiva Santos et al., 2012; Karimova et al., 2014). Different explanations about the increased risk of the RV failure in LVAD patients were proposed:

- RV work increase due to RV venous return increase thanks to the LVAD because the RV and the LV work are in series. The RV overload is proportional to the LVAD contribution (Mandarino et al., 1992; Omoto et al., 2002),
- the leftward shift of the interventricular septum after the LVAD implantation could decrease the support given by the septum to the RV contraction and the leftward shift is proportional to the LVAD contribution (Hendry et al., 1994; Omoto et al., 2002).

On the contrary, it has been considered that the LVAD could decrease the RV afterload, decreasing the pulmonary arterial pressure, possibly leading to a better RV pump functioning (**?**). In literature, there are different opinions and several authors performed also animal experiments to study the interventricular interaction during LVAD assistance (Kawai et al., 1992; Mandarino et al., 1992; Morita et al., 1992; Hendry et al., 1994; Chen et al., 1996; Barbone et al., 2001; Omoto et al., 2002; de Jonge et al., 2005; Kukucka et al., 2011; Saraiva Santos et al., 2012; Umeki et al., 2012; Arakawa et al., 2014; Karimova et al., 2014).

**Figure 1:**
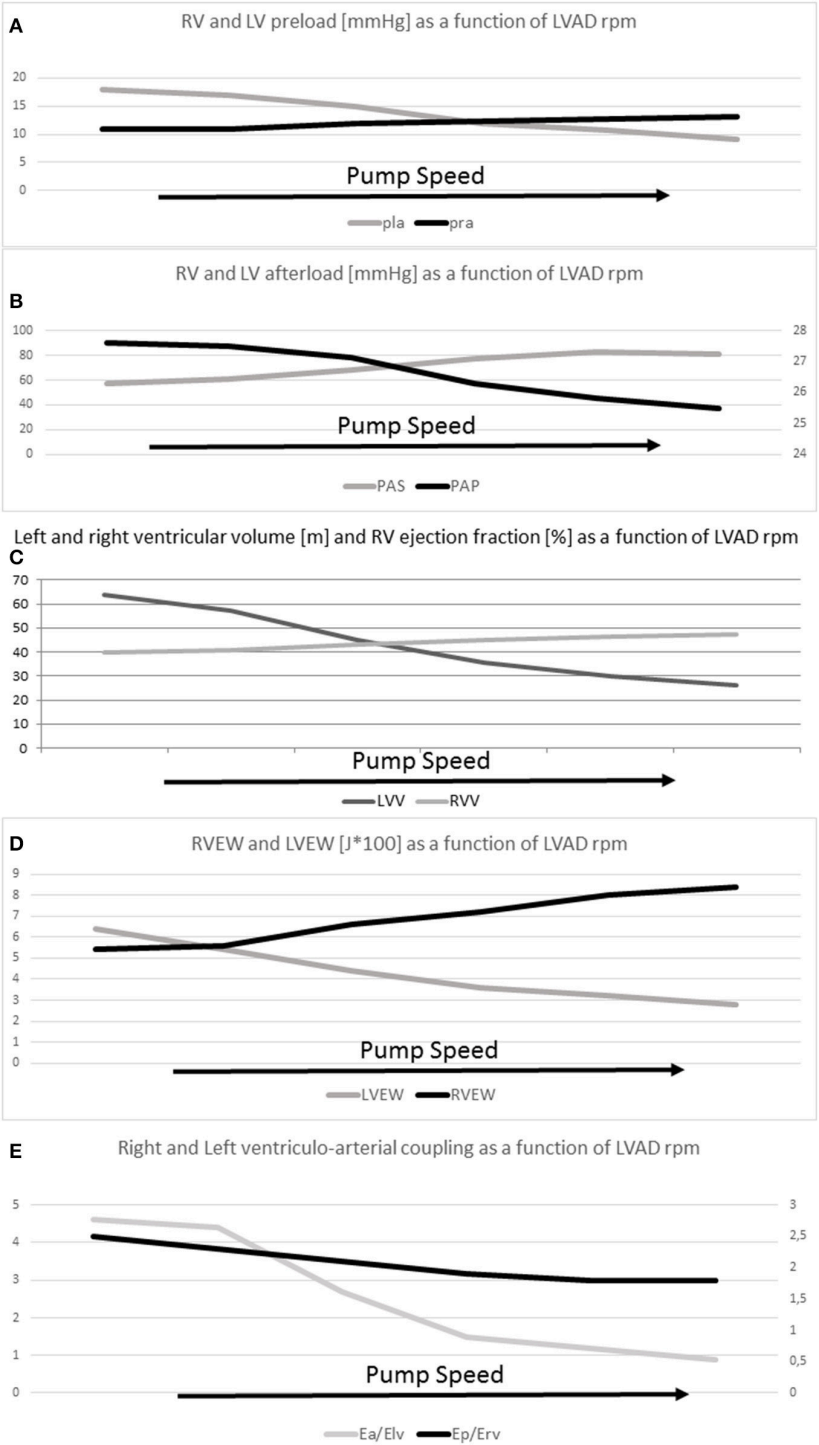
**(A)** RV (pra) and LV (pla) preload (mmHg), **(B)** RV (Pap) and LV (Pas) afterload (mmHg), **(C)** RV (RVV) and LV (LVV) average volumes (ml), **(D)** RV (RVEW) and LV (LVEW) external work (J^*^100), **(E)** RV (Ep/Erv) and LV (Ea/Elv) ventriculo-arterial coupling as a function of LVAD rate.

Di Molfetta et al. (2016) summarized with a simulation the effects of the LVAD implantation and the effects of the increased LVAD contribution using a lumped parameter model (Figure 1). Increasing the LVAD contribution a decrement of the LV preload and volumes and an increment of the arterial systemic mean pressure can be obtained together with an increment of the RV preload and RV volumes and a decrement of the pulmonary mean pressure. Finally, a decrement of the LV work and an increment of RV work are obtained increasing the pump contribution.

Analyzing the infant population, only the Berlin Heart EXCOR (BHE; Berlin Heart, Berlin Heart AG, Berlin, Germany) is licensed as long term VAD to bridge children to OHTx (Ibrahim et al., 2000; Reinhartz et al., 2003; Chang and McKenzie, 2005; Stiller et al., 2005; Hetzer et al., 2006; Gandhi et al., 2008; Brancaccio et al., 2010, 2013; Potapov et al., 2011; Almond et al., 2013; Miera et al., 2014). The smallest patients are particularly demanding in terms of morbidity and mortality (Ibrahim et al., 2000; Reinhartz et al., 2003; Chang and McKenzie, 2005; Stiller et al., 2005; Hetzer et al., 2006; Gandhi et al., 2008; Brancaccio et al., 2010, 2013; Potapov et al., 2011; Almond et al., 2013; Miera et al., 2014) especially now that the LVAD staying is increasing over time. In particular, small body surface area, BHE size mismatch and younger age seems to be associated with an increased risk of thromboembolic complications (Almond et al., 2013; Miera et al., 2014).

In a recent study, Conway et al. reported about the outcome of a multicenter study on 97 pediatric patients under 10 kg undergoing Berlin Heart EXCOR VAD. In their study, the median LVAD stay was 26 days with a survival to heart transplantation less than 60% (Conway et al., 2015).

On the basis of these considerations, the aim of this study is to retrospectively review our single-center experience on the use of BHE in children under 10 Kg.

## Materials and methods

### Patients' data collection

Data of all pediatric patients under 10 kg undergoing BHE implantation in our institution from March 2002 to March 2016 were retrospectively reviewed including:

- *Before the implantation*: gender, diagnosis, age at the implantation, weight at the implantation, previous surgery, comorbidities, requirement of ECMO;
- *During the implantation*: concomitant surgical procedure, requirement of biventricular assistance using two BHE, neurological complication, bleeding, cannulas infections, sepsis; BHE pump changes, length of VAD staying, clinical outcome (OHTx, death, heart recovery, still on VAD), and cause of death during the assistance;

Informed consent for the BHE implantation and the divulgation of clinical data was obtained by all patients' parents. The protocol was approved by the ethical board of our hospital. The protocol adheres to the principle expressed in the Declaration of Helsinki.

### Surgical procedure

All patients underwent BHE VAD implantation. The BHE consists of a paracorporeal, pneumatically driven, polyurethane blood pump with a multilayer flexible membrane separating the blood from the air chamber. Silicon cannulae connect the blood pump to the patient, and tri-leaflets inflow and outflow valves prevent blood reflux. All surface in contact with blood are heparin-coated. Each pump is driven by a pulsatile electro-pneumatic system. All BHE implantation were performed in a full median sternotomy and mild hypothermic cardiopulmonary bypass. The heart was kept beating throughout the procedure except in patients requiring the interatrial communication closure where ventricular fibrillation was used. Inflow cannulation for the left ventricular assist device (LVAD) was preferentially achieved through the left ventricular apex or, alternatively, through the left atrium (before 2007). The pump size was chosen according to the BHE indications (Miera et al., 2014). The VAD rate was set to assure an adequate perfusion ranging from 100 ml/kg/min to 200 ml/kg/min. All the other VAD parameters were set with the aim of assuring a full-empty full-fill operating modality and with the aim of assuring the aortic valve opening at least every three cardiac cycle.

### Anticoagulation strategy and patients monitoring

All patients were routinely evaluated performing one echocardiography every week. Concerning the anticoagulation strategy, in the first post-operative day, the unfractioned heparin was started at initial dose of 15 IU/Kg/h every 12 h and increased after 6 hours at therapeutic dose of 28 IU/Kg/h to obtain a PTTs between 1.5 and 2.5 times the basal value with a correspective anti Xa between 0.35 and 0.5. After 1 week, if the active bleeding was absent, the hemodynamic status was stable and renal function was normal, low molecular weight heparin (LMWH) was introduced at a starting dose of 150 IU/kg with a target anti Xa of 0.6–1.2 IU/ml. The anti-aggregation therapy was modulated by platelet mapping, between 2nd and 4th POD if bleeding was stopped. Dypiridamole was started at 1 mg/kg/day at the POD 4 while the aspirin was started at 1 mg/kg/day divided in 2 doses at the POD 7, when chest drain are removed. Oral anticoagulant drugs (warfarin) was considered in children older than 12 months when oral or enteral feeding is optimized. Initial loading dose was 0.2 mg/Kg/die (maximum dose of 5 mg/die) and target INR was between 2.7 and 3.5.

### Statistical analysis

Continuous variables are expressed as mean values and standard deviation or as median values, where appropiate. Survival analysis is performed by Kaplan-Meyer curves and log rank test (Mentel-Cox). Hazard ratio is estimated by Mentel-Haenszel test. Statistical analysis was performed using the commercial software MedCalc.

## Results

### Baseline data

A total of 30 patients under 10 kg were implanted from 2002 to 2016 in our institution. Sixteen children were male (53%) with a mean weight at the implantation of 6.75 ± 2.16 Kg and an average age at the implantation of 11.57 ± 10.12 months. Most of the patients (25/30, 87%) were affected by a dilated cardiomyopathy (secondary to myocarditis in 4 cases and with left ventricular non compaction in 3 cases), 2 (6.5%) patients were affected by a restrictive cardiomyopathy, 1 patient developed heart failure post cardiotomy and 2 (6.5%) patients had a failing univentricular heart physiology (Reinhartz et al., 2003). Before the VAD implantation, one patient underwent surgery for a supravalvular mitral ring and developed heart failure post cardiotomy and two patients underwent Glenn procedure. Before the BHE implantation, 8 patients (27%) were mechanically ventilated and 3 patients (10%) were receiving ECMO for cardiorespiratory failure (Table 1).

**Table 1:** **Patients Demographics**.

**Variables**	**Data**
Male/Female	16/14
Age at implant [months]	11.57 ± 10.12
Weight [Kg]	6.75 ± 2.16
**DIAGNOSIS**	
DCM	25 (83%)
RCM	2 (7%)
Failing UVH physiology	2 (7%)
After cardiotomy	1 (3%)

DCM, Dilatative Cardiomyopathy; RCM, Restrictive Cardiomyopathy; UVH, Univentricular Heart; VAD, Ventricular Assist Device.

### VAD implantation

Inflow cannulation for the LVAD was achieved through the left ventricular apex in the 27 (90%) children and through the left atrium in three. From August 2007, left atrial cannulation was dismissed in favor of left ventricular apical cannulation. An isolated LVAD was used in 27 (90%) patients and a BiVAD in three. All the BiVAD were implanted before 2007. Eight patients (27%) underwent concomitant atrial septal defect closure, while in one patient a ductus arteriosus was closed during the VAD implantation.

### Postoperative

Before 2007, two patients underwent percutaneous atrial septal defect closure with an Amplatzer device after the LVAD implantation for severe desaturation (Brancaccio et al., 2010). Seven patients (23%) required re-intervention for bleeding after the LVAD implantation and 9 patients (30%) experienced BHE cannulas infection. A total of 56 BHE pumps were changed along all the studied period for thrombus formation (1.86 BHE pumps for patient). Two LVAD patients underwent BHE pump upgrading from 10 ml to 15 ml because of patients weight increment after April 2013, when the 15 ml BHE became available.

### Outcome

The average duration of VAD support was 132.8 ± 94.4 days. Then, 20 patients (67%) were successfully transplanted and 10 patients (33%) died. Of these 10 patients, 7 died for major neurological complication and 3 for sepsis (Table 2).

**Table 2:** **Outcomes on VAD**.

**Outcome**	**No. (%) Tot No 30 patients**
Days of assistance	132.8 ± 94.4
OHTx	20 (67%)
Death on VAD	10 (33%)
Fatal neurological complications	7 (23%)
Fatal sepsis	3 (10%)
Cannulae infection	9 (30%)
Overall no of pump change	56 (1.86/pts)
Bleeding requiring surgery	7 (23%)

## Discussion

The use of BHE could successfully bridge pediatric patients till the OHTx, but the management of small patients is especially challenging. In fact, small body surface area seems to be associated with an increased risk of thromboembolic complications during VAD assistance and increased mortality as reported by Almond et al. (2013). In accordance with this consideration, Reinhartz et al. (2003) evidenced that an oversized device, which is simpler in smaller patients, could lead to an increased risk of thromboembolisms because of the stasis into the devices or systemic hypertension due to large stroke work. Similarly, Miera et al. (2014) demonstrated the influence of the BHE size in relation to patients weight on the clinical outcome. In fact, in their study Miera et al. evidenced that a large pump size in relation to body surface area is an independent risk factor for the occurrence of thromboembolic events (Miera et al., 2014). Moreover, they evidenced that younger age and smaller body surface area patients showed a trend of increased risk of thromboembolic events. In their work, Stiller et al. (2005) reported about the BHE implantation in 18 children less than 1 year underlying that the small children could be successfully bridged to OHTx, but the intensive care management and the anticoagulation strategy should be optimized to improve the outcome in terms of mobility and mortality. Gandhi et al. (2008) reported their experience on the use of BIVAD in 9 small children with an average weight of 9.4 kg (3 to 38 kg). Patients were supported for 35 days (1 to 77) and then 8 patients were successfully transplanted. It should be underlined that, as reported by Hetzer et al. (Potapov et al., 2011), the length of VAD staying is increasing over time and the management of small patients to achive long term event free VAD support is still challenging (Potapov et al., 2011). Finally, Conway et al reported about the outcome of a multicenter study on 97 pediatric patients under 10 kg undergoing Berlin Heart EXCOR VAD. In their study, the median LVAD stay was 26 days with a survival to heart transplantation less than 60% (Conway et al., 2015).

In our study, we reported our experience on the use of BHE in 30 patients under 10 kg. Patients were assisted for a median time of 132.8 ± 94.4 days that is a significantly longer time compared to the experiences reported in literature (Almond et al., 2013; Conway et al., 2015). Despite a longer period of support the complications and the outcomes of our population are comparable to the complications and the outcomes reported in literature. Sixty seven percentage of patients were successfully transplanted, 23% of patients required re-intervention for bleeding, 23% of patients experienced major neurological complication and 30% experienced BHE cannulas infection.

In conclusion, the use of BHE in small children is becoming of common use in recent years. Longer support time of several months requires a careful management of these fragile patients especially in terms of coagulation and infections. Good results in short and long term are possible, encouraging the use of this strategy as bridge to OHTx.

## Author contributions

FG, Clinical data; AD, Statistical analysis and clinical data; SF, Surgical procedure analysis; LD, Post operative data analysis; RI, Pre-op Echo analysis; RA, Preoperative analysis data; GP, Post-op Echo analysis; MD, Post-op Intensice care analysis; IF, Anticoagulation protocol; AR, Anticoagulant data analysis; GT, Post operative data analysis; AA, Paper Overview.

### Conflict of interest statement

The authors declare that the research was conducted in the absence of any commercial or financial relationships that could be construed as a potential conflict of interest.
